# Standardisation of prostate multiparametric MRI across a hospital network: a London experience

**DOI:** 10.1186/s13244-021-00990-y

**Published:** 2021-04-20

**Authors:** Marianthi-Vasiliki Papoutsaki, Clare Allen, Francesco Giganti, David Atkinson, Louise Dickinson, Jacob Goodman, Helen Saunders, Tristan Barrett, Shonit Punwani

**Affiliations:** 1 Centre for Medical Imaging, University College London, 2nd Floor Charles Bell House, 43-45 Foley Street, London, W1W 7TS UK; 2 Department of Radiology, University College London Hospital NHS Foundation Trust, Euston Road, London, WC1H 8NJ UK; 3 Division of Surgery and Interventional Science, University College London, 43-45 Foley Street, London, W1W 7TS UK; 4 North East London Cancer Alliance, Tower Hamlets CCG, London, E1 4DG UK; 5 North Middlesex University Hospital, Sterling Way, London, N18 1QX UK; 6 Department of Radiology, School of Clinical Medicine, University of Cambridge, Hills Road, Cambridge, CB2 0SP UK

**Keywords:** Multiparametric magnetic resonance imaging, Clinical protocols, Prostatic neoplasms, Diffusion, Contrast media

## Abstract

**Objectives:**

National guidelines recommend prostate multiparametric (mp) MRI in men with suspected prostate cancer before biopsy. In this study, we explore prostate mpMRI protocols across 14 London hospitals and determine whether standardisation improves diagnostic quality.

**Methods:**

An MRI physicist facilitated mpMRI set-up across several regional hospitals, working together with experienced uroradiologists who judged diagnostic quality. Radiologists from the 14 hospitals participated in the assessment and optimisation of prostate mpMRI image quality, assessed according to both PiRADSv2 recommendations and on the ability to “rule in” and/or “rule out” prostate cancer. Image quality and sequence parameters of representative mpMRI scans were evaluated across 23 MR scanners. Optimisation visits were performed to improve image quality, and 2 radiologists scored the image quality pre- and post-optimisation.

**Results:**

20/23 mpMRI protocols, consisting of 111 sequences, were optimised by modifying their sequence parameters. Pre-optimisation, only 15% of T2W images were non-diagnostic, whereas 40% of ADC maps, 50% of high b-value DWI and 41% of DCE-MRI were considered non-diagnostic. Post-optimisation, the scores were increased with 80% of ADC maps, 74% of high b-value DWI and 88% of DCE-MRI to be partially or fully diagnostic. T2W sequences were not optimised, due to their higher baseline quality scores.

**Conclusions:**

Targeted intervention at a regional level can improve the diagnostic quality of prostate mpMRI protocols, with implications for improving prostate cancer detection rates and targeted biopsies.

## Keypoints

- Standardisation of diagnostic image quality of prostate multiparametric MRI is crucial to optimise clinically significant prostate cancer detection.
- Pre-optimisation, the majority (85%) of the T_2_ -weighted images were partially or fully diagnostic, whereas 40% of ADC maps, 50% of high b-value diffusion-weighted images and 41% of dynamic contrast-enhanced MRI were non-diagnostic.
- After applying the standardisation process across the several prostate multiparametric MRI protocols, the majority of the scores were increased resulting in 80% of ADC maps, 74% of high b-value diffusion-weighted images and 88% of dynamic contrast-enhanced MRI to be partially or fully diagnostic.

## Introduction

Worldwide, there were an estimated 359,000 prostate cancer (PCa) deaths in 2018 [1]. The introduction of prostate multiparametric MRI (mpMRI) has revolutionised the management of PCa, improving diagnosis [2] and risk stratification of patients, and allowing appropriate subsequent management [3, 4]. In 2019, the National Institute of Health and Care Excellence (NICE) updated prostate cancer guidelines endorsing the routine use of mpMRI in biopsy-naive men with suspected PCa [5].

A range of challenges are evident in implementing prostate mpMRI nationally, many of which were discussed in the 2018 United Kingdom (UK) Prostate Cancer Consensus Meeting [6]. Several studies have highlighted that mpMRI quality varies substantially between centres and scanners, which is vulnerable to patient-related degradations, and that poor image quality is associated with greater uncertainty and lower accuracy [7–9]. Subsequently, the acquisition of mpMRI images of good diagnostic quality is crucial. Without this, any interpretations made by radiologists (no matter how experienced) is likely to be flawed and could subsequently lead to incorrect patient management.

Prostate mpMRI consists of 3 components: T_2_-weighted (T2W) anatomical imaging, diffusion-weighted MRI (DW-MRI) assessment of tissue cellularity, and dynamic contrast-enhanced MRI (DCE-MRI) evaluation of tissue vascularity. Prostate Imaging and Reporting and Data System [10, 11] and UK Consensus meetings [6, 12, 13] have provided written guidance on imaging protocol set-up. However, there is currently no system in place whereby centres perform a formal quality check of their mpMRI scans to confirm diagnostic acceptability. Furthermore, smaller centres may have less experienced radiographers, generalist rather than specialist reporting radiologists and absence of MRI physicist support, and therefore they are more likely to have non-optimised protocols.

This study explores regional prostate mpMRI protocols across 23 MR scanners situated across 14 London hospitals by assessing their diagnostic quality and determining whether standardisation can improve their diagnostic quality.

## Materials and method

**Fig. 1:**
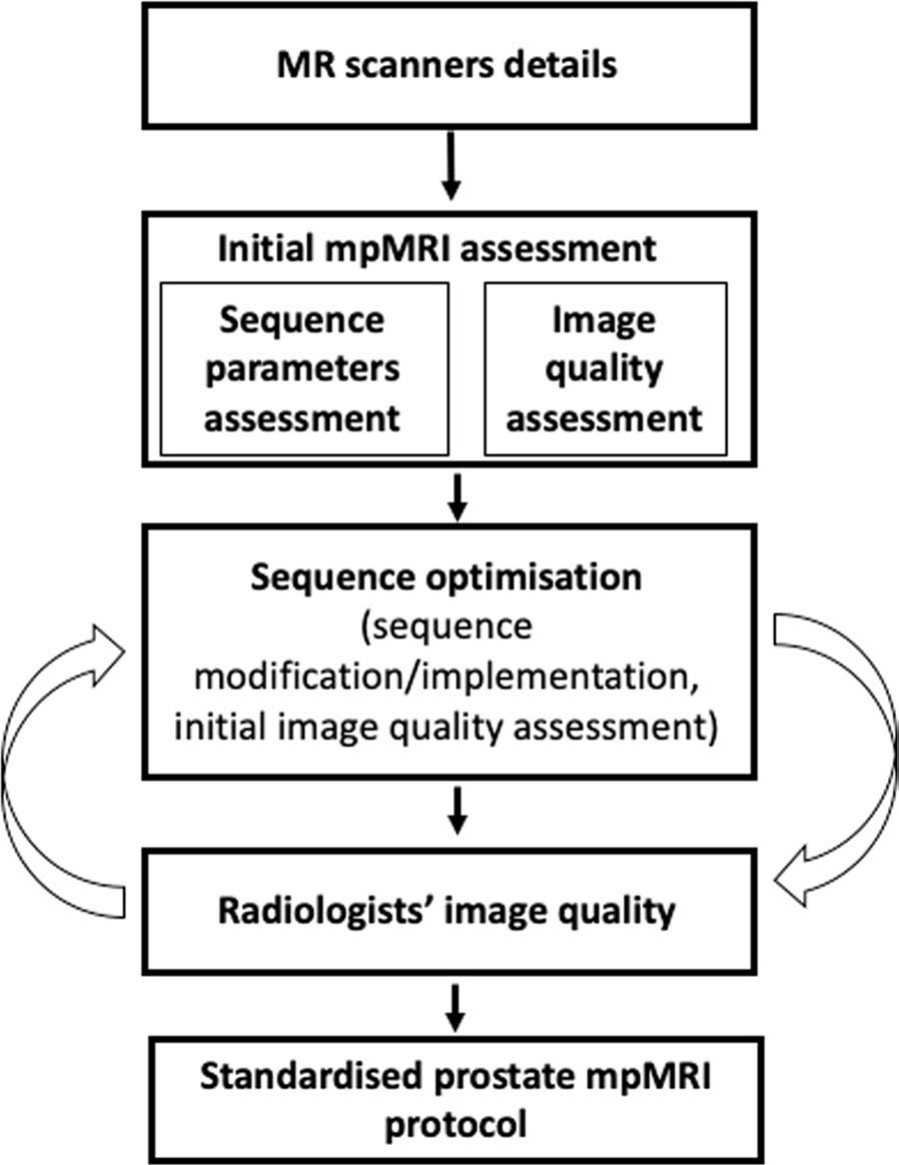
Flow chart presenting the outline of the optimisation procedure of the prostate multiparametric (mp) MRI protocols

An MRI physicist (M.-V.P.) experienced with prostate mpMRI (2 years) was employed by North Central and East London Cancer Alliance in a dedicated role to facilitate mpMRI set-up across the regional network hospitals. The physicist led the optimisation of mpMRI protocols over a year (from May 2018 to April 2019, based on the availability of time for optimisation at each hospital), working closely with 2 uroradiologists from the region leading hospital (C.A., L.D., each with > 5 years prostate mpMRI and reporting > 500 studies per year). In Fig. 1, the flow chart provides an outline of the optimisation process.

### Optimisation set-up

In June 2018, radiologists specialised in reporting prostate mpMRI across different hospitals in North Central and East London Cancer Alliance network were invited to participate in a prostate imaging meeting. The aim of the meeting was to identify the need of acquiring acceptable diagnostic quality prostate mpMRI, to define an image quality system for prostate mpMRI and to invite them to participate in the set-up and use of a standardised and diagnostic quality protocol for prostate mpMRI. Subsequently, 20 radiologists from 14 hospitals (totally 23 MR scanners) participated (7 hospitals had 2 MR scanners and one had 3 scanners). These hospitals work independently in terms of prostate mpMRI, but their relationship with the leading hospital is that all the prostate cancer surgery is performed at the leading hospital. Hence, it would be important all of them to adopt the same imaging set-up ensuring high diagnostic quality of the prostate mpMRI. Therefore, a quality assurance framework for prostate mpMRI was proposed and accepted by the participants, aiming to establish a reliable imaging set-up and protocol across the region. The imaging set-up and protocol were determined following PiRADSv2 recommendations which was current at the time the work commenced [10]. Based on PiRADSv2, required sequences for prostate mpMRI protocol were: (a) multiplanar T2W, (b) two DW-MRI sequences (one for the apparent diffusion coefficient (ADC) map production and another for the high b-value diffusion-weighted imaging (DWI) and (c) one DCE-MRI sequence. The diagnostic image quality standards were defined based on the ability to “rule in” and “rule out” PCa [14]. According to recently proposed PI-QUAL scoring system [14], a 5-point scoring system was used for each required sequence, assigning: a) low score (1 or 2) as non-diagnostic, when it was not possible to either “rule in” or “rule out” PCa, b) medium score (3) as partially diagnostic, when it was possible to “rule in" but not possible to “rule out” PCa, and c) a high score (4 or 5) as fully diagnostic, when it was possible to both “rule in” and “rule out” PCa.

Scanner characteristics, patient set-up and acquired sequences were recorded for each one of the protocols. A representative prostate mpMRI from each MR scanner was assessed for sequence parameters by the physicist compared to PiRADSv2 standards and for diagnostic quality by the two uroradiologists using the above 5-point scoring system.

### Optimisation procedure

Following the review of MRI scans, visits for protocol optimisation were organised. The optimisation involved the modification of sequence parameters according to PiRADSv2 recommended sequence parameters and radiologists’ scores. During the optimisation, images from the pre-optimised (original) protocol were acquired on real-life patients of each hospital list undergoing prostate mpMRI, followed by optimised sequences (patients were consented for longer protocol duration). At the end of the optimisation session, an initial informal review of quality assessment was performed by at least two radiologists, one from the leading and one or more from the visiting hospital, on the optimised sequences. Further visits were performed if the images were considered not yet fully optimised. Once completed, the new optimised protocol was integrated by the hospital. Due to the high baseline quality scores of T2W images, the DW-MRI and DCE-MRI protocols were prioritised for the optimisation process. For the DCE-MRI protocol optimisation, it was not possible to inject the patient twice. We acquired the pre-optimised sequence during contrast injection and the new sequence at time points just before and just after this in order to compare images with similar amounts of injected contrast in the tissue. When an improvement in the image quality was observed for the optimised sequence, that protocol was examined during the contrast agent injection and chosen as the final optimised.

### Formal image review

The prostate MR images of PiRADSv2 required sequences were qualitatively assessed by two radiologists in consensus from the leading hospital, one with 20 years (C.A.) and another with 7 years (F.G.) reading experience. Five image acquisitions were reviewed: (1) axial T2W, (2) coronal T2W, (3) sagittal T2W, (4) ADC map, (5) high b-value DWI and (6) DCE-MRI. For the majority of the scanners, DW-MRI and DCE-MRI sequences were acquired twice on the same patient, with the pre- and post-optimised sequences. The radiologists were unaware of which sequence was pre- and which was post-optimisation. For each anonymised sequence, a qualitative assessment was performed (Table 1) followed by an overall diagnostic acceptability using the pre-optimisation 5-point scoring system.

**Table 1:** Diagnostic quality assessment questionnaire

	T_2_-Weighted			Diffusion-weighted imaging (DWI)		Dynamic contrast-enhanced MRI
	Axial	Coronal	Sagittal	DWI	High b-value DWI	
Angulation	Yes/no	Yes/no	Yes/no	Yes/no	Yes/no	Yes/no
Does the current angulation match with the T2W axial?				Yes/no	Yes/no	Yes/no
Image resolution	Poor/adequate/good	Poor/adequate/good	Poor/adequate/good	Poor/adequate/good	Poor/adequate/good	Poor/adequate/good
FOV	Small/sufficient/large	Small/sufficient/large	Small/sufficient/large	Small/sufficient/large	Small/sufficient/large	Small/sufficient/large
SNR	Low/adequate/high	Low/adequate/high	Low/adequate/high	Low/adequate/high	Low/adequate/high	Low/adequate/high
b-values (s/mm^2^)				0, 150, 500, 1000	1400 @ 1.5 T 2000 @ 3.0 T	
Artifacts	Yes/no	Yes/no	Yes/no	Yes/no	Yes/no	Yes/no
Image blurring due to motion	Yes/no	Yes/no	Yes/no	Yes/no	Yes/no	Yes/no
Is it possible to rule in tumours?	Yes/no	Yes/no	Yes/no	Yes/no	Yes/no	Yes/no
Is it possible to rule out tumours?	Yes/no	Yes/no	Yes/no	Yes/no	Yes/no	Yes/no
Is it possible to clearly visualise the periprostatic and cavernosal vessels?						Yes/no
Number of dynamic measurements						Few/adequate/many
Temporal resolution of each dynamic measure						$\le \hspace{0.17em}$10 s

## Results

**Fig. 2:**
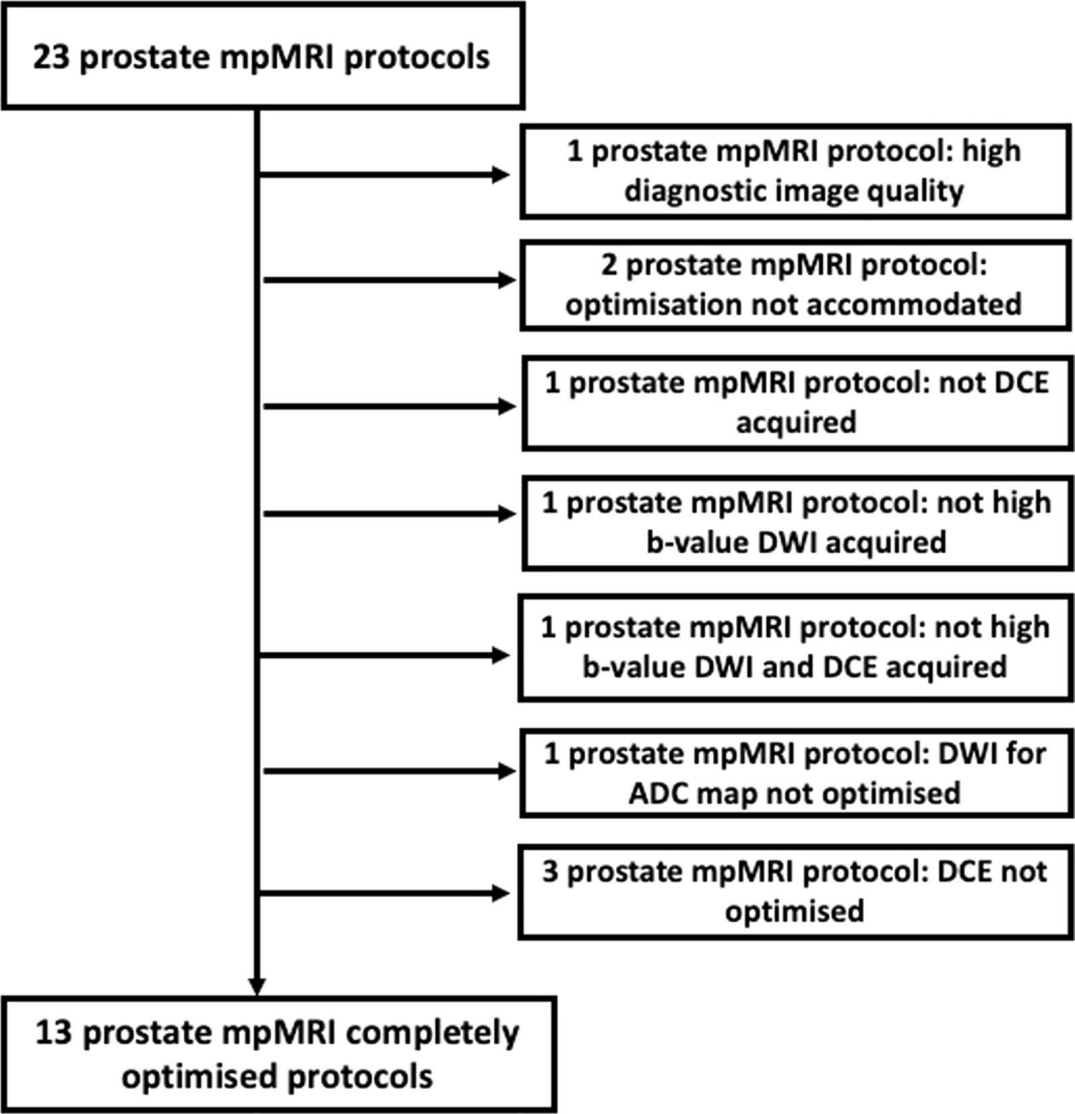
Flow chart presenting the initial number of assessed prostate multiparametric (mp) MRI protocols and the final number completely optimised acquiring all the recommended sequences. (DWI: diffusion-weighted imaging, DCE: dynamic contrast enhanced)

From 23 prostate mpMRI protocols, one presented high diagnostic image quality and was used as an exemplar (Fig. 2). Two protocols were not optimised, because the optimisation visits could not be carried out. Subsequently, 20 protocols were optimised, consisting of 111 sequences, acquired by MRI scanners aged from 1 to 16 years old (1/20 MRI scanner was 16 years old, 3/20 were 14 years old, 4/20 were 9 years old, 5/20 were 8 years old, 2/20 was 5 years old and 5/20 were 1 year old) from different manufacturers (7/20 S AG, Erlangen Germany, 7/20 GE Healthcare Waukesha, WI and 6/20 Philips Healthcare, Best, The Netherlands); 4/20 operated at 3.0 T and 16/20 at 1.5 T.

### Pre-optimisation patient set-up and imaging protocol

Patient set-up was evaluated in terms of patient position, coil and administration of antispasmodic agent. Only in 1/20 (5%) protocol, patient’s position was headfirst. 15/20 (75%) protocols utilised surface phased array coils and 5/20 (25%) used body array coils. For 6/20 (30%), an antispasmodic agent was administered prior to imaging.

2/20 (10%) protocols acquired only axial and coronal T2W images, which was compliant with the newer PiRADSv2.1 recommendations, but not with the PiRADSv2. The other protocols acquired T2W across the 3 orthogonal orientations. 14/20 (70%) protocols acquired a high b-value DWI acquisition, where 2/14 (14.3%) utilised the calculated high b-value DWI. 3/20 (15%) protocols did not include DCE-MRI. The mean protocol duration was 33 min (range 18–45 min). For all protocols, the sequence parameters of the reviewed sequences were evaluated against PiRADSv2 and PiRADSv2.1 standards (Table 2).

**Table 2:** Summary of acquisition parameters range prior the optimisation related to Prostate Imaging Reporting and Data System (PiRADSv2 and v2.1)

Sequence parameter	PiRADSv2 standard (PiRADSv2.1 standard)	Mean	Range
*Axial T*_*2*_*-weighted*			
Field of view (mm)	120–200	200	170–240
Slice thickness (mm)	3	3.2	3–4
Slice gap (mm)	0	0.3	0.-0.75
Acquired pixel size (mm) × (mm)	≤ 0.4 (frequency)	0.7 (frequency)	0.5–1.0
	≤ 0.7 (phase)	0.9 (phase)	0.6–1.3
*Coronal T*_*2*_*-weighted*			
Field of view (mm)	120–200	204	180–240
Slice thickness (mm)	3	3.1	3–4
Slice gap (mm)	0	0.3	0–0.7
Acquired pixel size (mm) × (mm)	≤ 0.4 (frequency)	0.7 (frequency)	0.5–1.0
	≤ 0.7 (phase)	0.9 (phase)	0.6–1.1
*Sagittal T*_*2*_*-weighted*			
Field of view (mm)	120–200	212	180–250
Slice thickness (mm)	3	3.5	3–6
Slice gap (mm)	0	0.4	0–2
Acquired pixel size (mm) × (mm)	≤ 0.4 (frequency)	0.8 (frequency)	0.6–1.1
	≤ 0.7 (phase)	1.0 (phase)	0.6–1.5
*Diffusion-weighted MRI (DW-MRI)*			
Repetition time (ms)	≥ 3000	4921	1320 -23,651
Echo time (ms)	≤ 90	80	48–117
Field of view (mm)	160–220	275	220–380
Slice thickness (mm)	≤ 4	4.45	2.5–6
Slice gap (mm)	0	0.5	0–1
Acquired pixel size (mm) × (mm)	≤ 2.5 (frequency)	2.23	1.3–4.8
	≤ 2.5 (phase)	2.64	1.5–4.8
Number of b-values for ADC map	At least 2 b-values	3.35	2–4
Proposed b-values (s/mm^2^)	(0), 50, 150, 500, 1000		
High b-value (s/mm^2^)	1400 at 1.5 T	1333	1200–1500
	2000 at 3 T	1400	1200–1600
*Dynamic contrast-enhanced MRI (DCE-MRI)*			
Repetition time (ms)	< 100	5.4	3.2–8.2
Echo time (ms)	< 5	2.25	1.6–3.2
Field of view (mm)	Encompass the entire prostate gland and the seminal vesicles	274	205–400
Slice thickness (mm)	3	3	2–6
Slice gap (mm)	0	0.8	0–3
Acquired pixel size (mm) × (mm)	≤ 2.0 (frequency)	1.4	0.75–1.65
	≤ 2.0 (phase)	1.5	0.75–2.07
Temporal resolution (s)	$\le$ 10 ($\le$ 15)	14.7	6–32
Total duration	≥ 2 min	5 min	2 min–10 min 46 sec

### Post-optimisation patient set-up and imaging protocol

Only 1 sequence was optimised in 2/20 mpMRI protocols, 2 sequences in 6/20, whereas 3 sequences in 12/20. In total, 50/57 (88%) sequences were either optimised or implemented including 18/20 (90%) sequences for ADC map production, 19/20 (95%) for high b-value DWI, and 13/18 (72%) for DCE-MRI. The PiRADSv2 patient set-up was recommended, included feet first for patient’s comfort, surface coil and administration of an antispasmodic agent. 2/5 protocols adopted the cardiac coil and 5/14 protocols the administration of an antispasmodic agent. 3/20 (15%) hospitals did not have a cardiac or surface coil. 3/20 (15%) protocols included the high b-value DWI with a 5 min increase in protocol duration and 1/3 protocol included DCE-MRI. The mean protocol duration following optimisation was 33 min (range 21–43 min).

All DW-MRI sequences used the same b-values for the ADC map, the spatial resolution and FOV all complied with PiRADSv2 but with a 5 mm slice thickness. For the high b-value DWI, all the 1.5 T sequences encompassed the b-value of 1400 s/mm^2^ and all the 3.0 T protocols a b-value of 2000 s/mm^2^ and thus were compliant with PiRADSv2. In DCE, the slice thickness, the spatial resolution adhered to PiRADSv2, the temporal resolution was longer but then adhered to PiRADSv2.1.

### Overall diagnostic acceptability per imaging protocol

**Fig. 3:**
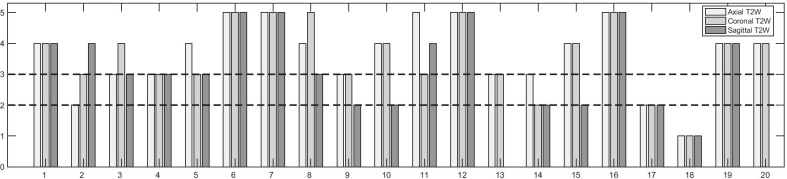
Scores of the image quality assessment of the T_2_-weighted (T2W) (axial, coronal and sagittal) images. The numbers denote the MR scanners

**Fig. 4:**
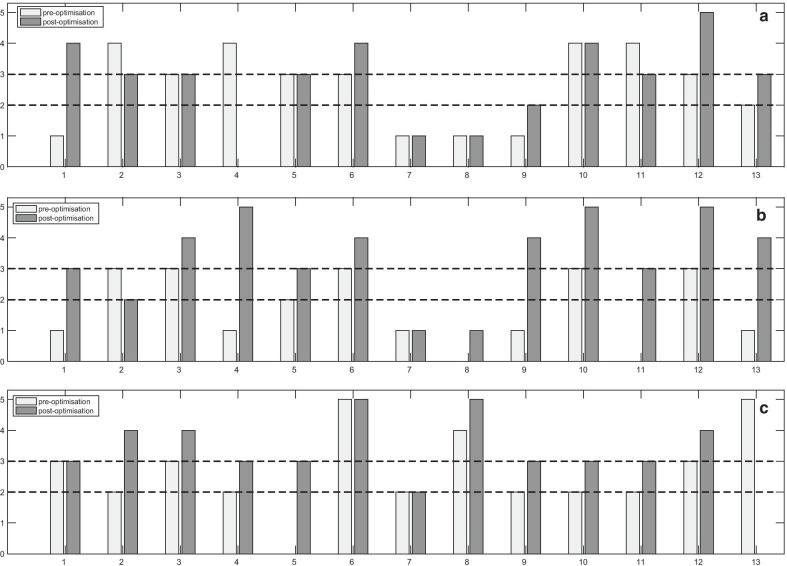
Pre- and post-optimisation scores of the completely optimised multiparametric MRI protocols of (**a**) apparent diffusion coefficient (ADC) maps, (**b**) high b-value diffusion-weighted (DW) images and (**c**) dynamic contrast-enhanced (DCE) MRI. The numbers denote the MR scanners

**Fig. 5:**
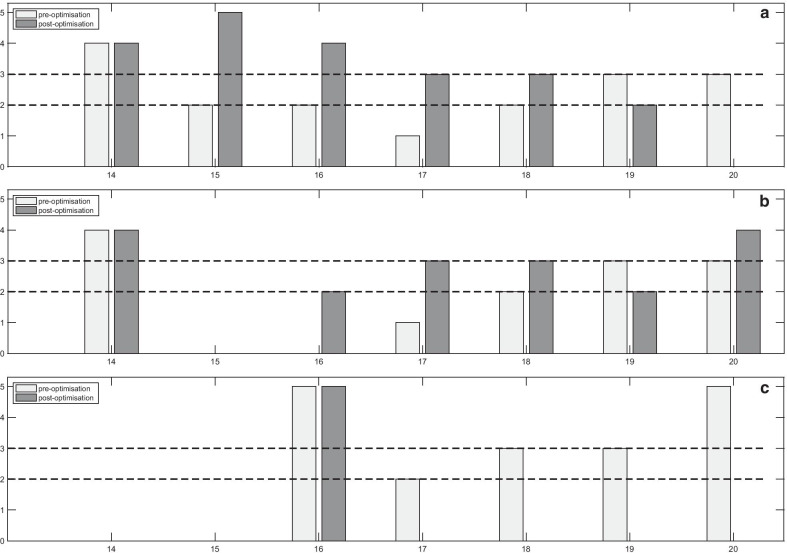
Pre- and post-optimisation scores of the incompletely optimised multiparametric MRI protocols of (**a**) apparent diffusion coefficient (ADC) maps, (**b**) high b-value diffusion-weighted (DW) images and (**c**) dynamic contrast-enhanced (DCE) MRI. The numbers denote the MR scanners

In 13/20 mpMRI protocols, both DW-MRI and DCE-MRI sequences were completely optimised and complied with PiRADSv2, whereas in 7/20 protocols it was not possible for all sequences to be fully optimised (Fig. 2). The scores per sequence pre- and post-optimisation for the completely and incompletely optimised protocols are presented in Figs. 3, 4 and 5.

After the optimisation, the number of fully and partially diagnostic sequences was increased. Post-optimisation, 9/39 (23%) DW-MRI sequences were scored as non-diagnostic. In all of these cases, the age (> 8 years old) of the MR scanners restricted the optimisation process and necessitated longer protocol durations for full implementation of PiRADSv2 recommendations. The corresponding hospitals were unable to accommodate any further increase in protocol duration due to scheduling constraints.

### Image quality of T2-weighted images

For the majority of the protocols, the T2W sequences were partially or fully diagnostic (Fig. 3). For the axial T2W sequences, 3/20 (15%) sequences were non-diagnostic, 5/20 (25%) were partially, and 12/20 (60%) were fully diagnostic. For the coronal T2W, 3/20 (15%) sequences were non-diagnostic, 6/20 (30%) were partially, and 11/20 (55%) were fully diagnostic. For the sagittal T2W, 3/18 (16.7%) sequences were non-diagnostic, 7/18 (38.9%) were partially, and 8/18 (40%) were fully diagnostic. Non-diagnostic T2W sequences were associated with poor image resolution and low signal-to-noise ratio (SNR) (acquired pixel size of 0.8 to 1.5 mm across the frequency encoding (FE) and the phase encoding (PE) direction). Good image resolution was associated with an acquired pixel size of 0.7 mm across the FE and PE directions. Optimisation of the T2W sequences on early hospital visits made no measurable difference to quality scores (in 6/20 (30%) scanners), and due to time constraints, priority was subsequently given to DWI and DCE on further visits.

### Image quality of apparent diffusion coefficient (ADC) maps

**Fig. 6:**
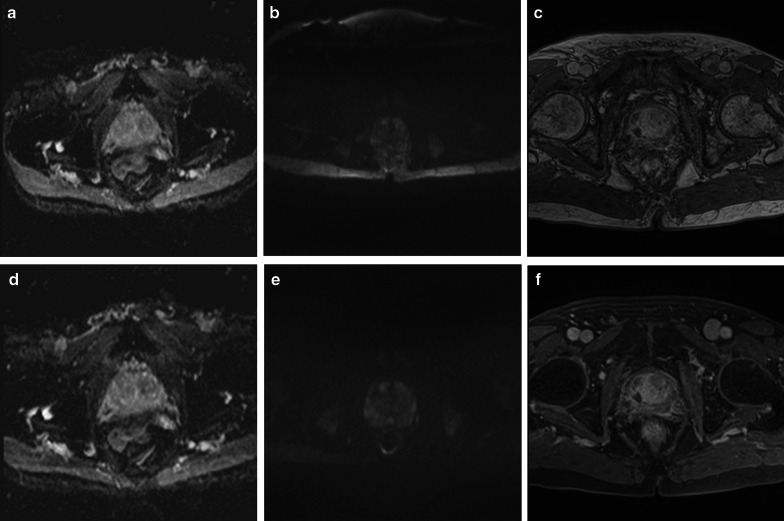
Images (**a**–**c**) acquired pre-optimisation, showing: **a** apparent diffusion coefficient (ADC) map with large field of view; **b** high b-value diffusion-weighted (DW) image with large field of view and chemical shift artefact; **c** dynamic contrast-enhanced (DCE) image without fat suppression technique, 30th measurement post-contrast. Following optimisation, images (**d**–**f**) demonstrated the changes in the image quality at the same patient: **d** ADC map with optimal field of view; **e** high b-value diffusion-weighted (DW) image with optimal field of view and without chemical shift artefact; **f** DCE image with fat suppression technique, 3rd measurement post-contrast

Pre-optimisation, the scores of the ADC maps (Figs. 4a, 5a) showed that 8/20 (40%) sequences were non-diagnostic, 7/20 (35%) were partially, and 5/20 (25%) were fully diagnostic. Non-diagnostic ADC maps were associated with large FOVs (ranged from 250 to 380 mm) (Fig. 6a), poor image resolution (acquired pixel size ranged from 2.6 mm to 4.8 mm across FE and PE directions) and low SNR (slice thickness smaller than 5 mm, with less than 3 b-values in combination with the number of signal averages (NSA)).

Post-optimisation, 18/20 (90%) sequences were modified, resulting in improvement from 4/18 (22.2%) to 7/18 (38.9%) fully diagnostic, from 6/18 (33.3%) to 7/18 (38.9%) partially diagnostic and a reduction from 8/18 (44.4%) to 4/18 (22.2%) non-diagnostic ADC maps. For some MR scanners, longer protocol duration was required for improved diagnostic quality. For example: for the SNR increase, the number of signal averages (NSA) had to be increased resulting in a longer protocol duration; for image resolution improvement, the voxel size had to be reduced by increasing the number of phase encoding lines in the acquisition matrix, which increased further the protocol duration. However, these hospitals were unable to increase their protocol duration despite the suggestions; subsequently, some ADC maps remained non-diagnostic after the optimisation. Figure 6d shows an example of an ADC map with optimal FOV post-optimisation, as compared with the pre-optimisation large FOV acquired from the same patient (Fig. 6a).

### Image quality of high b-value diffusion-weighted images

Pre-optimisation, 16/20 protocols included the acquisition or the calculation of the separate high b-value DWI (Figs. 4b, 5b). 8/16 (50%) sequences were non-diagnostic, 7/16 (43.8%) were partially, and 1/16 (6.2%) was fully diagnostic. Non-diagnostic high b-value DWI was associated with large FOV, poor image resolution and low SNR. The range of the large FOV and the acquired pixel size resulting in poor image resolution was the same as the ADC maps (Fig. 6b). Low SNR was reported for slice thickness smaller than 5 mm with few NSA, ranged from 2 to 8, and small acquired pixel size, ranged from 1.3 mm to 2.5 mm.

Post-optimisation, 16/20 (75%) high b-value DW sequences were optimised and another 3 were implemented, resulting in improvement from 1/16 (6.2%) to 9/19 (47.7%) fully diagnostic, a reduction from 7/16 (43.8) to 5/19 (26.3%) partially diagnostic and from 8/16 (50%) to 5/19 (26.3%) non-diagnostic high b-value DWI. An example of an optimised high b-value DW image is presented in Fig. 6e, where an optimal FOV without chemical shift artefact is illustrated post-optimisation, as compared to the large FOV with the chemical shift artifact on pre-optimisation image for the same patient (Fig. 6b).

### Image quality of dynamic contrast-enhanced MRI

17/20 (85%) prostate mpMRI protocols included DCE-MRI (Figs. 4c, 5c). 7/17 (41.1%) DCE-MRI sequences were non-diagnostic, 5/17 (29.4%) were partially and 5/17 (29.4%) fully diagnostic. The non-diagnostic sequences were associated with poor image resolution, (resulted by a thick slice ($\ge \hspace{0.17em}$3 mm) or a large acquired pixel size ranged from 1.48 to 2.02 mm), large FOV (ranged from 260 to 400 mm) and low SNR (depicted to acquired pixel size, ranged from 0.88 to 1.65 mm or to a slice < 3 mm). Figure 6c presents a poor image quality DCE acquisition without a fat suppression technique.

12/20 (60%) DCE sequences were optimised and one new sequence was implemented. Post-optimisation, the scores were improved from 3/12 (25%) to 6/13 (46.1%) fully diagnostic, from 3/12 (25%) to 6/13 (46.1%) partially diagnostic and a reduction from 6/12 (50%) to 1/13 (7.7%) non-diagnostic DCE-MRI. Figure 6f presents a DCE image post-optimisation with fat suppression technique of the 3rd post-contrast measurement, as compared to the pre-optimisation image without fat suppression for the same patient (Fig. 6c).

## Discussion

In the current study, prostate mpMRI protocols across 23 MR scanners situated across 14 London hospitals were explored, and after standardisation, the overall diagnostic quality was improved in the majority. The initial protocol heterogeneity, in terms of patient set-up, sequence type and parameters, was reduced after the optimisation resulting in a common and standardised procedure. Post-optimisation, the diagnostic acceptability of mpMRI was improved, increasing radiologists’ confidence in “ruling in” and “ruling out” PCa. Other multicentre studies highlighted the need for prostate mpMRI optimisation not only to comply with PiRADSv2 [7, 15], but also to produce scans with adequate diagnostic quality [9]. Jorge et al. [16] introduced the role of director of imaging for standardising prostate mpMRI performance. Five mpMRI protocols of 4 institutions were assessed and modified only according to PiRADSv2 standards, without examining their diagnostic image quality. To our knowledge, there is no other study assessing and optimising mpMRI protocols across different hospitals according to PiRADSv2 standards and their diagnostic image quality pre- and post-optimisation.

PiRADSv2 standards were used for study design and optimisation. These standards were updated to PiRADSv2.1 during the course of the study. For example, although 3 orthogonal planes were required for PIRADSv2 for the T2W images, PiRADSv2.1 only requires axial and one additional plane. In general, PiRADSv2.1 criteria were less restrictive than PiRADSv2. However, it should be mentioned that compliance with PiRADS guidelines does not necessarily equate to good image quality [8], for some scanners maintaining technical compliance reduced diagnostic quality. It should be mentioned that we included in the optimisation the DCE-MRI, despite the fact that its inclusion is controversial in prostate mpMRI. Currently there is not enough evidence to confirm its exclusion.

This study aimed at improving image quality using the existing scanner resources at each of the 14 hospitals. Post-optimisation, the practice at 5/14 (36%) hospitals was changed to include the use of antispasmodics and 1/3 hospital added a DCE protocol. The diagnostic acceptability was improved. 14% sequences for the ADC map production became fully diagnostic (totally 40%), 40% were partially diagnostic and 20% non-diagnostic. Regarding the high b-value DWI, 41.2% became fully diagnostic (totally 47.4%) and 26.3% remained partially and 26.3% non-diagnostic. 17% DCE-MRI became fully diagnostic (totally 47%), 41% were partially and 12% non-diagnostic. These results depict the various challenges during the optimisation.

The major challenge was to perform the optimisation in men undergoing prostate mpMRI within the routine schedule of each hospital. This was achieved by adding 10 min to each prostate mpMRI scan, enabling the acquisition of pre- and post-optimised sequences for each patient. Several iterations of post-optimised sequences were required in order to achieve best quality images, best achieved over several different scans in order that the length of scan for each patient was not excessive. This approach required the booking of at least 4 prostate mpMRI scans during each optimisation session, although this was difficult to schedule for the majority of the hospitals. For at least one of the booked scans, either the patient did not attend the session or had MR contraindications or was unable to tolerate the scan any longer. In some cases, the anatomical factors (e.g. after radical prostatectomy) did not allow the optimisation to be carried out. Other "real-life" limitations included the presence of rectal air, deteriorating the optimisation in DW-MRI sequences, especially where no antispasmodic agent was administered [17]. It is known that the different magnetic susceptibilities of prostate tissue and rectal air introduce magnetic field inhomogeneities which can result in distortions or in signal voids at the posterior part of prostate [18].

Another limiting factor was in the different capabilities of the MR scanners, which varied in field strength, manufacturer, age, hardware and software. For old (age > 8 years) 3.0 T scanners, the optimisation of the DW-MRI sequences was more challenging, as compared to the new (1 year) 3.0 T scanners. In patients’ scans where no antispasmodic agent was administered and the rectum was full of air, distortions and signal voids were more prominent at 3.0 T due to the magnetic field inhomogeneities, as compared to the 1.5 T scanners. At high b-value DW-MRI at 3.0 T, the suggested b-value (b = 2000 s/mm^2^) required higher NSA to increase SNR and image quality [6, 10]. However, this as well as the increase of spatial resolution and SNR led to a longer acquisition time, which was not possible in some hospitals in terms of workload capacity, resulting in non-diagnostic sequences post-optimisation (scanners 8 and 16). These hospitals mentioned that a longer protocol duration would have an impact on their clinical scheduling. Either the radiographers would work out of hours or less patients would be scanned daily. At 1.5 T scanners, the age and the different software also influenced the optimisation. In older scanners (scanners 2, 11 and 19), the recommended reduced FOV in combination with an adequate image resolution in DW-MRI resulted in a remarkable reduction in SNR, although this could not be justified by the significant increase in acquisition time. Indeed, time constraints were a major restriction on sequence optimisation due to scheduling constraints typical of busy imaging services. For the majority of the hospitals, three to four visits were needed for a completed optimisation for all the sequences. However, it was not feasible for many hospitals to accommodate more than one visit in their daily clinical schedule. Ideally, optimisation should be considered an ongoing process, and with planned scanner updates, upfront optimisation will further improve network standards and diagnostic quality. Lastly, the majority of the hospitals did not include the use of any antispasmodic agent initially. However, during the course of the optimisation, some hospitals used an antispasmodic agent as a test. The radiologists of these observed the positive impact on the image quality of this agent; subsequently, they decided to adopt it in their clinical practice.

Our study has some limitations. The optimised sequences could only be acquired and scored only in one patient; subsequently, we recognised that the image quality assessment was also dependant on patient’s body habitat. Ideally scans should be acquired on many more patients and scored both pre- and post-optimisation. Moreover, the pre- and post-optimised DCE-MRI sequences were acquired and compared either pre- or post-injection, because it was not feasible to inject the same patient twice. For few protocols, the DCE-MRI was not optimised, and this was due to limited time for optimisation at the particular hospitals. Lastly, the overall impact of the optimisation on diagnosis and management was not assessed, due to the fact that it was beyond the study scope and duration.

This study explored regional prostate mpMRI protocols across 23 MR scanners situated across 14 London hospitals, demonstrated heterogeneity in diagnostic quality and showed how targeted intervention could help standardise and improve diagnostic quality. We show a methodology for engagement of non-specialist hospitals, show which scans typically are problematic and quantify how much of a change this type of intervention can achieve. Although populations and the management of healthcare differs by region, the information presented should be informative for many settings. This work presents as an example the prostate mpMRI standardisation across a hospital network in London. Other hospitals or countries might adopt a different approach depending on their regulations and their clinical schedule.

## Data Availability

The data are included in this manuscript.

## Abbreviations

ADC: Apparent diffusion coefficient
DCE-MRI: Dynamic contrast-enhanced magnetic resonance imaging
DW-MRI: Diffusion-weighted magnetic resonance imaging
FE: Frequency encoding
FOV: Field of view
mpMRI: Multiparametric magnetic resonance imaging
NICE: National Institute of Health and Care Excellence
NSA: Number of signal averages
PCa: Prostate cancer
PE: Phase encoding
PiRADS: Prostate Imaging and Reporting and Data System
SNR: Signal-to-noise ratio
T2W: T_2_-weighted

## References

[1] Culp MBB, Soerjomataram I, Efstathiou JA (2020). Recent global patterns in prostate cancer incidence and mortality rates. Eur Urol.

[2] Ahmed HU, El-Shater Bosaily A, Brown LC (2017). Diagnostic accuracy of multi-parametric MRI and TRUS biopsy in prostate cancer (PROMIS): a paired validating confirmatory study. Lancet.

[3] Simmons LAM, Kanthabalan A, Arya M (2017). The PICTURE study: diagnostic accuracy of multiparametric MRI in men requiring a repeat prostate biopsy. Br J Cancer.

[4] Kasivisvanathan V, Rannikko AS, Borghi M (2018). MRI-targeted or standard biopsy for prostate-cancer diagnosis. N Engl J Med.

[5] National Institute for Health and care Excellence. Prostate cancer: diagnosis and management. NICE guideline. www.nice.org.uk/guidance/ng131. 35263066

[6] Brizmohun Appayya M, Adshead J, Ahmed HU (2018). National implementation of multi-parametric magnetic resonance imaging for prostate cancer detection—recommendations from a UK consensus meeting. BJU Int.

[7] Esses SJ, Taneja SS, Rosenkrantz AB (2018). Imaging facilities’ adherence to PI-RADS v2 minimum technical standards for the performance of prostate MRI. Acad Radiol.

[8] Sackett J, Shih JH, Reese SE (2020). Quality of prostate MRI: is the PI-RADS standard sufficient?. Acad Radiol.

[9] Burn PR, Freeman SJ, Andreou A (2019). A multicentre assessment of prostate MRI quality and compliance with UK and international standards. Clin Radiol.

[10] Weinreb JC, Barentsz JO, Choyke PL (2016). PI-RADS prostate imaging-reporting and data system: 2015, version 2. Eur Urol.

[11] Turkbey B, Rosenkrantz AB, Haider MA (2019). Prostate imaging reporting and data system version 2.1: 2019 update of prostate imaging reporting and data system version 2. Eur Urol.

[12] Kirkham APS, Haslam P, Keanie JY (2013). Prostate MRI: who, when, and how? Report from a UK consensus meeting. Clin Radiol.

[13] de Rooij M, Israël B, Tummers M (2020). ESUR/ESUI consensus statements on multi-parametric MRI for the detection of clinically significant prostate cancer: quality requirements for image acquisition, interpretation and radiologists’ training. Eur Radiol.

[14] Giganti F, Allen C, Emberton M (2020). Prostate imaging quality (PI-QUAL): a new quality control scoring system for multiparametric magnetic resonance imaging of the prostate from the PRECISION trial. Eur Urol Oncol.

[15] Cuocolo R, Stanzione A, Ponsiglione A (2019). Prostate MRI technical parameters standardization: a systematic review on adherence to PI-RADSv2 acquisition protocol. Eur J Radiol.

[16] Abreu-Gomez J, Shabana W, McInnes MD, O’Sullivan JP, Morash CSN (2019). Regional standardization of prostate multiparametric MRI performance and reporting: is there a role for a director of prostate imaging?. AJR Am J Roentgenol.

[17] Caglic I, Barrett T (2019). Optimising prostate mpMRI: prepare for success. Clin Radiol.

[18] Caglic I, Hansen NL, Slough RA (2017). Evaluating the effect of rectal distension on prostate multiparametric MRI image quality. Eur J Radiol.

